# Co-creation of practical “how-to guides” for patient engagement in key phases of medicines development—from theory to implementation

**DOI:** 10.1186/s40900-021-00294-x

**Published:** 2021-08-23

**Authors:** David Feldman, Paola Kruger, Laure Delbecque, Ashley Duenas, Oana Bernard-Poenaru, Séverine Wollenschneider, Nick Hicks, Janine Ann Reed, Ify Sargeant, Chi Pakarinen, Anne-Marie Hamoir, on behalf of Patient Focused Medicines Development Working Groups 1, Oana Bernard-Poenaru, Katherine Deane, David Feldman, Grace Fox, Gorbenko Oleksandr, Jim Hartke, Nick Hicks, Vivian Larsen, Benjamin Missbach, Claire Nolan, Natasha Ratcliffe, Carole Scrafton, Merlin Williams, on behalf of Patient Focused Medicines Development Working Groups 2A, Ashley Duenas, Dagmar Kaschinski, Dominique Hamerlijnck, Janelle Goins, Janet Peterson, Jessica Scott, Laure Delbecque, Paola Kruger, on behalf of Patient Focused Medicines Development Working Groups 2B, Adit Bassi, Angi Gillen, Duane Sunwold, Janine Ann Reed, Jeanette Ryan, Jennifer Preston, Marta Garcia, Olga Zvonareva, Rob Camp, Ronella Grootens, Severine Wollenschneider, Thierry Escudier

**Affiliations:** 1 National Kidney Foundation, New York, NY USA; 2 REPE—Rete Pazienti Esperti, Roma, Italy; 3 Eli Lilly Benelux N.V, Brussels, Belgium; 4 Evidera, London, UK; 5 Institut de Recherches Internationales Servier, Suresnes, France; 6 F. Hoffmann-LaRoche, Basel, Switzerland; 7 Commutateur Advocacy Communications, Paris, France; 8 National Kidney Foundation, Alport Syndrome Foundation, New York, NY USA; 9 Twist Medical, Burlingame, CA USA; 10 The Synergist, Brussels, Belgium

**Keywords:** Practical guidance patient engagement medicines development

## Abstract

**Background:**

The effective impact of patient engagement (PE) across the medicines development continuum is widely acknowledged across diverse health stakeholder groups, including health authorities; however, the practical applications of how to implement meaningful and consistent PE are not always addressed. Guidance for the practical implementation of PE requires granularity, and the need for such guidance has been identified as a priority. We describe the co-production and summarize the content of how-to guides that focus on PE in the early stages of medicines development.

**Methods:**

Multi-stakeholder working groups (WGs) were established by Patient Focused Medicines Development (PFMD) for how-to guide development. How-to guides were co-produced with patients for PE activities identified as priorities through public consultation and by WGs. Guides were developed by applying PE quality guidance and associated quality criteria in an iterative process. How-to guides underwent internal review and validation by experts (ie, those with relevant experience in the particular PE activity or focus area) in specific focus groups and external review and validation through appropriate events and public consultation.

**Results:**

Overall, 103 individual contributors from 38 organizations (representing eight stakeholder groups, including patients/patient organizations) and from 14 countries were organized into WGs and workstreams. Each WG comprised 15–30 contributors with PE experience relevant to the specific how-to guide. How-to guides were developed for PE in the early discovery and preclinical phases; PE in the development of a clinical outcomes assessment strategy; and PE in clinical trial protocol design. The how-to guides have a standardized format and structure to promote user familiarity. They provide detailed guidance and examples that are relevant to the individual PE activity and aim to facilitate the practical implementation of PE.

**Conclusions:**

The how-to guides form a comprehensive series of actionable and stepwise resources that build from and integrate the PE quality criteria across the medicines continuum. They will be made freely available through PFMD’s Patient Engagement Management Suite (pemsuite.org) and shared widely to a variety of audiences in different settings, ensuring access to diverse patient populations. Implementation of these guides should advance the field of PE in bringing new medicines to the market and ultimately will benefit patients.

**Plain English summary:**

Medicines are developed to help patients improve their health and lives. Many organizations and individuals want to ensure that medicines are developed to meet real patient needs and to address what is most important to patients. Finding out what patients need and what patients want requires good patient engagement, but knowing *how* to do patient engagement is not always clear. This is because medicines development is complicated, and a lot of different steps, people, and organizations are involved. Patient Focused Medicines Development (PFMD) was established in 2015 to connect individuals and organizations that are committed to making medicines not just *for* patients but *with* patients. To do this, PFMD brought together patients and other groups of people with relevant experience and good ideas on how to achieve patient engagement in the real-world setting. Together, PFMD has developed “how-to guides” for patient engagement that cover the main activities along the medicines development process. The guides are free to use and provide practical advice and examples that anyone can use in their patient engagement activities. The how-to guides will also help patients to understand medicines development and how best they can participate in this process to address their needs.

**Supplementary Information:**

The online version contains supplementary material available at 10.1186/s40900-021-00294-x.

## Introduction

The essential role of patient engagement (PE) across the medicines development continuum is widely acknowledged across diverse health stakeholder groups. Regulatory, access, and health technology assessment (HTA) stakeholders recognize the need and importance of partnering with patients – as the ultimate end-users of products and solutions – during their decision-making processes. Collaborations between such agencies have been established to increase the integration of the patients’ perspective into their regular activities. Effective, collaborative, and early integration of the patient voice in medicines development by the pharmaceutical industry is becoming the expected norm and, in some instances, a requirement [1, 2].

An increasing number of efforts to deliver meaningful PE have been documented [3–11]. Importantly, a range of tangible benefits have been reported for development programs that incorporate PE, including improved recruitment and retention, as well as faster study completion [12]. There are also published guidance, frameworks, and tools for PE [13–18], some of which focus on specific phases of the medicines lifecycle, such as clinical trials and research [5, 19–26] or a particular disease or condition [25, 27–29]. Regulators and HTA/payers have also provided guidance and frameworks on how to best engage patients in medicine development and regulatory decision making [30–32]. A common theme for this shift toward more consistent PE is the importance of multi-stakeholder co-creation to ensure that solutions and outputs are relevant, meaningful, and valuable for all patients. However, there remains an unmet need for detailed guidance to support PE in core activities across the entire medicines development pathway.

Patient Focused Medicines Development (PFMD) was established in October 2015 as an open, independent, global coalition of diverse health stakeholder representatives, including patients, with the common goal of integrating the patient voice in the design and development of research and medicines [33, 34]. A core focus of PFMD activity is to synergize disparate, but complementary, efforts to co-create a comprehensive framework for impactful and consistent PE that comprises guidance, tools, and resources and that spans the medicines development continuum and beyond (e.g., medical devices industry, care). PFMD has established a rational and stepwise approach to collaborative co-production of this framework [35], as previously described in detail [16].

While the need for PE is accepted, the practical applications of how to do it are not always addressed, and definitions, meaning, and processes for PE vary. PE quality guidance was co-created to provide practical advice and criteria to facilitate the implementation of meaningful PE [16]. The PE quality guidance provides an important and widely applicable foundation for PE. However, guidance for implementation of PE for a *specific* activity requires additional detail. This is only achievable through a good understanding of the environment in which the PE is to happen and the associated best practices that allow challenges to be anticipated and addressed. The need for this tailored and specific guidance is evidenced by the increase in documentation of individual PE projects that provide the level of detail needed to guide others wanting to replicate or conduct similar PE activities [36–39].

Despite this encouraging increase, there remains a largely unmet need for practical “how-to” guidance on specific PE activities that are relevant across health stakeholder groups (including patients, industry, HTA, and others). Such guidance was identified as a priority in a global, multi-stakeholder consultation [40]. In particular, there is a lack of guidance and resources for PE in the early stages of medicines development. Since many health stakeholders believe that PE should begin as early as possible at the start of the medicines development process [16, 33, 34, 41, 42], PFMD WGs have co-produced how-to guides for incorporating PE in the early phases of medicines development. In this paper, we describe the co-production of these guides, building on past experience with the development of PE quality guidance [16] and summarizing the content in each guide. These cover early discovery and preclinical phases, clinical outcome assessment (COA) measurement strategy, and clinical trial protocol design. The how-to guides form an integral part of the Patient Engagement Management (PEM) Suite, an aggregation of existing knowledge, experts and expertise, actionable tools, and good practices for PE in medicines development.

## Methodology

### Identification and prioritization of PE activities for guidance co-creation

As part of the process for development of the PE quality guidance [16], a landscape analysis was undertaken to identify PE activities across the phases of medicines development. In addition, a public consultation was conducted for validation and prioritization of identified PE activities to guide the focus of future PE implementation efforts. The methodology and results of the public consultation and prioritization have been previously reported [40]. Briefly, an online consultation was conducted between June and August of 2018. Overall, 133 respondents completed the 22-question survey to prioritize 94 unique PE activities. PE activities that were identified as priorities through this public consultation were reviewed and further prioritized by WGs based on their expertise, relevant experience, and insights in PE, as well as their insights in medicines development phases and processes. How-to guides were co-created for prioritized PE activities.

### Establishment of the network of co-creation contributors

PFMD called for interested individuals to join the international co-creation multi-stakeholder WGs by communicating about the WGs in the PFMD membership and network from June through October 2018 via snowball technique. WG contributors were required to have PE experience and expertise in one or more phases of medicines development. An open webinar was held in December 2018 to provide additional information, and final WGs were formed in January 2019. Work on the how-to guides was initiated in February 2019. Different WGs developed each how-to guide, and contributors were invited to join a group based on how their specific experience related to the focus of the respective how-to guide. Details of WG contributors in the core team (defined as those who were actively involved in most aspects of each activity from conceptualization and design to co-development and delivery) are listed in the Supplementary Material, Table S1. WG contributors include core team members of the WG and reviewers (defined as those who reviewed and provided feedback on draft guidance/outputs and/or participated in review rounds).

### How-to guide co-creation

**Fig. 1:**
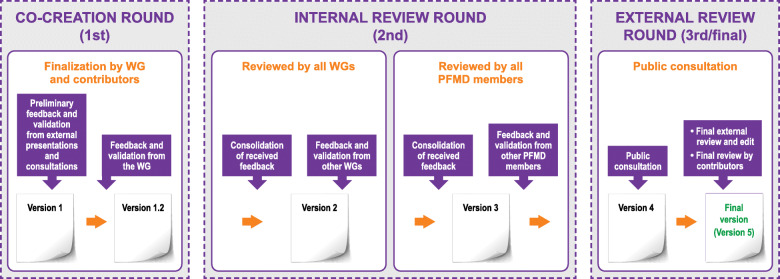
Feedback and validation process for how-to guides

Multi-stakeholder WGs co-produced their how-to guide according to the PE quality guidance [16]. The process is summarized in Fig. 1. Briefly, WGs developed the preliminary content and structure of the how-to guide during workshops, which was presented to a wide audience at the PE Open Forum in 2019 and 2020 (held in Brussels, Belgium, on September 18–19, 2019, and as a series of virtual meetings from June to November in 2020). The forums were organized by PARADIGM (Patients Active in Research and Dialogues for an Improved Generation of Medicines), PFMD, and EUPATI (European Patients’ Academy on Therapeutic Innovation). PE Forum delegates in 2019 participated in 10 interactive workshops, three of which were focused on co-creating and providing feedback on the how-to guides (Fig. 1; co-creation round). The draft guides were then reviewed by all the WGs as well as the PFMD network and all feedback received was aggregated and reviewed by the relevant WG for that guide. All feedback was considered and discussed in collaborative meetings. Individual WG members volunteered to address any feedback in their area of ‘expertise’ or experience, especially where additional work was needed to address the feedback. All feedback that was agreed by WGs to be relevant was incorporated into the next version of the guide (Fig. 1; internal review round). A final review was conducted through public consultation (via online survey and supported with social media outreach and a communication campaign to encourage participation) to capture wider feedback outside of the WGs and PFMD networks (Fig. 1; external review round).

### How-to guide review and validation

The how-to guides underwent three rounds of review and validation by WGs and the PFMD network, by expert reviews in specific focus groups, by participants in appropriate external events (e.g., specialized conferences), and through public consultation. How-to guides were refined following each review.

## Results

### Identification of PE activities and prioritization through public consultation

The prioritization of PE activities through public consultation [40] and the core team review led to the identification of activities at specific phases of medicines development as well as crosscutting topics relevant across all phases. Most responses were received for PE activities in research, discovery and early development, and in preclinical and clinical phase 1–3 trials. Activities rated to be particularly relevant in these phases included the use of patient experience to design research methodology, PE in trial design and operations, and PE in the development of outcome measures [40]. Consequently, and following further prioritization by WG contributors, three WGs focused their efforts on co-production of how-to guides for these PE activities (Table 1). Other WGs focused on PE activities in the regulatory phase, post-launch phase, and the co-creation of plain language summaries (not described in this paper). The public consultation also showed that the greatest number of priority activities were taking place in clinical phase 1–3 trials. Based on member experience and interest, WG2 focused on two priority activities: PE in COA measurement (WG2A), and PE in clinical trial protocol design (WG2B).

**Table 1:** Summary of how-to guides for PE in early phases of medicines development and contributors

How-To Guide	Summary	Contributors		
		Core Team (N)	Stakeholder Groups Represented in the Core Team	Reviewers (N)
PE in the early discovery and preclinical phases (WG1)	Includes practical guidance on how to: ● Identify key patient partners to gain important insights that help improve early research by identifying and focusing on unmet needs ● Gather patient input to satisfy these needs ● Incorporate PE in developing research methodology ● Foster valuable and long-term partnerships	13	● Patient/advocate/organization/association (*n* = 4) ● Pharmaceutical industry (*n* = 4) ● Academia/researchers (*n* = 3) ● Clinical research organization (CRO)/other service provider (*n* = 2)	31
PE in the development of a COA strategy (WG2A)	Includes practical guidance on how to: ● Engage patients and caregivers to establish a patient-focused COA strategy *This guide is relevant for phase I–III clinical trials*	8	● Patient/advocate/organization/association (*n* = 2) ● Pharmaceutical industry (*n* = 5) ● CRO/other service provider (*n* = 1)	11
PE in clinical trial protocol design (WG2B)	Includes practical guidance on how to: ● Integrate the patient perspective in protocol design and development *This guide is relevant for phase I–III clinical trials*	12	● Patient/advocate/organization/association (*n* = 3) ● Pharmaceutical industry (*n* = 5) ● Academia/researchers (*n* = 3) ● CRO/other service provider (*n* = 1)	N/A*

^*a*^*Contributors include core team members of the WG (defined as those who were actively involved in most aspects of each activity from conceptualization and design to co-development and delivery) and reviewers (defined as those who reviewed and provided feedback on draft guidance/outputs and/or participated in review rounds)*

*Review will take place in Q2 2021

### Co-creation contributor network

**Fig. 2:**
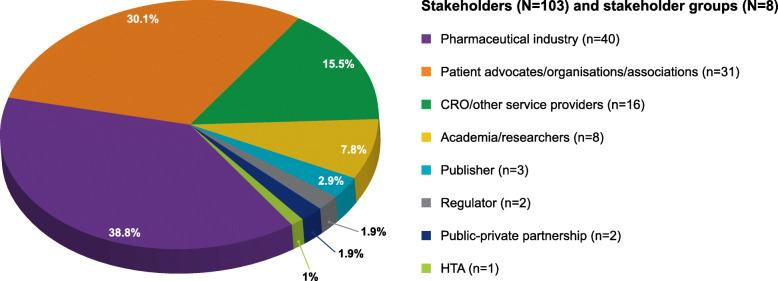
Multi-stakeholder composition of co-creation contributors network

A total of 103 individual contributors from 38 organizations were organized into WGs and workstreams. Among all the contributors, approximately a third (31 of 103 contributors; 30.1%) were patients, patient organization representatives, or patient advocates. Of these 31 contributors, seven were people with lived experience and were also members of patient organizations. Contributors represented eight different health stakeholder groups (Fig. 2) across 14 countries.

The composition of WG1, WG2A, and WG2B is summarized in Table 1 (also refer to Supplementary Material, Table S1) and included core team contributors, as well as reviewers (*N* = 75). Reviewers for each WG were drawn from the contributors network and included people with lived experience. Each WG comprised a range of stakeholders with PE experience relevant to the specific how-to guide or workstream.

### How-to guide co-production, review, and validation

How-to guides were co-produced in parallel following a similar process (Fig. 1) and were reviewed and refined at several milestones (summarized in Table 2). Currently two how-to guides are in public consultation.

**Table 2:** How-To Guide co-production milestones

	How-To Guide for PE		
Co-production milestones	Early discovery and preclinical phases (WG1)	Development of a COA strategy (WG2A)	Clinical trial protocol design (WG2B)
Version 1: Drafting of initial scope and structure by WG	March–August 2019	June–August 2019	June–August 2019
Version 1.2: Refinement following internal WG review	Shared for feedback at PE Open Forum September 2019	Shared for feedback at PE Open Forum, September 2019, and during a standalone working session held in parallel to the International Society for Pharmacoeconomics and Outcomes Research Europe, November 2019	Shared for feedback at PE Open Forum, September 2019
Version 2.0: Refinement following review and feedback	January–June 2020 Shared for feedback at PE Open Forum, June 2020	January–August 2020 Shared for feedback at PE Open Forum, September 2020	October 2019–ongoing
Version 3: Refinement following internal WG and contributor network review	July–November 2020	October–December 2020	
Version 4: Public consultation	December 2020–January 2021 Presented at Patients as Partners, January 2021	January 2021–ongoing	Planned ~April/July 2021

### How-to guides: format and structure

The how-to guides have a consistent format and structure that promotes user familiarity and ease of navigation. This is to facilitate the use of as many guides as relevant for any PE initiative and across the entire medicines development continuum. In general, each how-to guide begins with a descriptive overview to provide the user with background and context and a high-level understanding of where the guide fits in the PE landscape. This is followed by a brief description of the rationale and scope of the guide, including for whom it is intended and how it can be used. The majority of each document focuses on providing guidance and relevant examples specific to that activity and with the level of detail and hands-on descriptions required for the implementation of PE. Most guides also include annexes and links to relevant resources and tools, as well as a glossary or terminology section and a reference list of key publications.

### How-to guide for PE in the early discovery and preclinical phases

The how-to guide for PE in the early discovery and preclinical phases of medicines development focuses on practical elements and guidance along each step of the PE journey through the early discovery and preclinical phases. It contains four main steps (summarized in Table 3).The section “Preparations for setting up partnership and collaboration” describes considerations for patients and patient groups (such as managing expectations around the length of time that preclinical research takes and the potential need for prioritization of research questions) and for both patients and research teams (such as resources and time required and other logistics, preparations and practicalities that are prerequisite for in-person meetings). There is also guidance around general points to consider for patients and research teams that reflects PE quality criteria [16], such as defining collaborations and agreement of common goals and achieving clarity on roles and responsibilities. The first section aligns across all how-to guides because it focuses on the importance of partnership, which is relevant to all PE activities in medicines development.

**Table 3:** Summary of how-to guide for PE in the early discovery and preclinical phases

Section	Description and purpose/rationale
STEP 1: Preparations for setting up partnership and collaboration	*Focuses on the importance of preparations to ensure that long-term partnerships are created and nurtured. This sets the stage for future collaborations and aims to prevent tokenistic and one-off patient engagement.* ● Understand patients’ views and abilities to contribute to initial steps of drug development for the condition ● Prepare patients for participation in the program by educating patients on what preclinical research is ● Ensure that research-active organizations (e.g., academia and the pharmaceutical industry), regulators, governments, and other health system entities recognize and accept the importance of patient input in early medicines development ● Prepare the research team for a meaningful, effective, and respectful interaction with patients and give them tools to build a mutually beneficial long-term partnership
STEP 2: Understanding the condition profile and therapy area	*Reveals to researchers and scientists the patients’ experience and perspective on what it is like to live with the condition under study and what might be the unmet needs* ● Gather patient input to educate preclinical scientists/researchers about: the patients’ symptom burden and impact on daily life; what therapies patients use and their associated efficacies (or lack); patients’ views on an ideal therapy; patients’ views on risk-benefit trade-offs they will accept with new treatments ● Uncover patients’ unmet needs ● Engage and help patients to understand what preclinical research is, why it is needed, and where it sits in the overall medicines development continuum ● Act as an initial dialogue between patients, research teams, and other stakeholders toward creating a long-term relationship across the span of the medicine’s development
STEP 3: Developing research methodology	*Focuses on working with patients to evaluate and identify the optimal tools and approaches to address research objectives* Describes how to ● Work with patients to evaluate optimal tools and approaches to address research objectives in laboratory-based and virtual (in silico) studies ● Work with patients to evaluate possible studies to address clinical questions ● Define a range of outcome measures to be used in upcoming clinical studies ensuring these are patient-centered, and understand how outcome measures in laboratory-based and preclinical studies will translate into clinical outcomes that are meaningful for patients ● Consider public health implications and real-world anecdotal evidence in the research
STEP 4: Target product profiles and target value profiles	*Brings together outcomes from previous sections to develop two profiles that represent (1) the patients’ perspective on the product and (2) researcher/pharma-oriented documents that support research and development (R&D)* ● A target value profile (TVP) is a consolidated set of “expected and minimally acceptable characteristics” of a chemical molecule, biological product, or medical device, which are used as treatments and are valuable and meaningful for patients because they address areas of unmet needs. Along with other insights, the TVP informs the target product profile (TPP)—an updatable guidance for the pharma industry/drug developers with targeted characteristics of a potential product. The TVP—as a main element of TPP that encompasses core values and addresses unmet patient needs—should be co-developed with patients ● The guide provides examples of how to gain patient input and incorporate patients’ value in the TVP

The section “Understanding the condition profile and therapy area” focuses on giving researchers and scientists real-life patient perspectives of the condition under study and provides practical advice for defining collaboration goals; identifying potential patient partners; selecting and inviting patients to collaborate; helping patients learn about preclinical research; developing questions with patients to capture patient experiences; gathering patient input through forums and other methods; gap analysis; and priority setting. The section “Developing a research methodology” describes preparations specific to, as well as key activities relevant to, this stage, including possible topics for discussion. The section “Developing the target product/value profile” describes the expected characteristics of a potential product and the related desired value to be delivered to patients. It also explains how to involve patients in developing a relevant target product/value profile that contains their opinions, needs, and preferences, and also provides practical examples of questions that may facilitate co-production.

### How-to guide for PE in COA strategy development

The how-to guide for PE in COA strategy development describes a rational stepwise approach for achieving PE in this specific activity (summarized in Table 4). The first step, “*Preparations for setting up partnership and collaboration*,” incorporates guidance on general considerations, as well as those for the patient community and for medicine developers when establishing effective relationships. In fact, it draws widely on the PE quality guidance [16]. The other six steps follow a uniform structure and have common elements. First is a description of the step and then guidance on how to engage (methodology). This includes qualitative (such as patient/carer interviews, focus groups, and advisory board meetings) and quantitative approaches (such as patient and carer surveys or questionnaires), as well as mixed method research (a combination of qualitative and quantitative approaches). The next elements cover who is engaged (e.g., individual patients, carers, patient experts), what information is to be provided (e.g., disease-specific input; input on feasibility for clinical trial participants to complete the COA as described in clinical trial protocols; feedback on existing COAs). Also covered is when in the drug development process (e.g., preclinical development, clinical development, regulatory submission, and post-launch activities) these pursuits should be undertaken.

**Table 4:** Summary of how-to guide for PE in the development of a COA strategy

Section	Description and purpose/rationale
STEP 1: Preparation for partnership and collaboration with patients	*Focus is on engaging patients, establishing relationships, understanding patients’ views on participating, and defining the expectations of all parties* Prepares stakeholders for participation in the program by: ● Educating patients on COA instruments and strategy (to the required level depending on anticipated input) ● Educating the study team on the importance of patient input in the development of a COA strategy ● Preparing the study team for a meaningful, effective, and respectful interaction with patients and giving them tools to build a mutually beneficial relationship ● Establishing the foundation for a long-term, collaborative patient–sponsor relationship
STEP 2: Identification of relevant concepts (i.e., the burden of disease)	*Starting point of any COA strategy* ● Gaining a clear understanding from the patient (and/or carer) of their experience with the disease (and its treatment) and its impact on their life and well-being ● Identification and understanding of the signs, symptoms, and treatment burden patients experience and how these affect patients’ day-to-day functioning and quality of life to inform the COA strategy and ensure that the selected COA instrument(s) include(s) concepts that are relevant and important to patients
STEP 3: COA selection	*Undertaken after identifying concepts that are clinically relevant, important for patients, and likely to be affected by the drug* Describes how to secure patient input and collaboration to: ● Review existing COAs to determine which measure(s) provides the best insight(s) into the key domains of interest ● Determine the relevance of the measure(s) for use within a clinical trial ● Assess if the measure(s) is/are appropriate for its intended use and context of use (e.g., measurement properties, targeted study design and objectives, and targeted patient population)
STEP 4: COA development/revisions	*Undertaken if the evaluation done under Steps 2 and 3 reveals that no existing measure meets the selection criteria that are relevant to the target population and context of use* ● Describes how to secure patient input and collaboration to develop new COA tools or modify existing ones in accordance with available guidelines to meet regulatory requirements
STEP 5: COA implementation within clinical trials	*Patients’ roles in reviewing the trial protocol and advising on elements, including (but not limited to)* ● COA end points ● Feasibility for trial participants to complete the COA as described in the protocol ● Information provided to the trial participants with regard to the rationale for completing the COA instrument and how/when these data will be used
STEP 6: COA data interpretation	*Collecting patient insights to provide a valuable perspective on what constitutes clinically meaningful change for COAs* ● Conduct qualitative studies (e.g., in-trial exit interviews), surveys, or stakeholder meetings to discuss clinically meaningful change ● Patient experts involved in the study may provide input and facilitate communication with stakeholders to support the interpretation and importance of patient-relevant end points
STEP 7: COA communication	*Communicating COA data to augment limited product labeling and potentially hard to access scientific communication* ● Incorporating PE to improve the clarity and meaningfulness of communications around COA data by providing input (e.g., development, review, approval) on the information that will be disclosed before its publication

### How-to guide for PE in clinical trial protocol design

The how-to guide for PE in clinical trial protocol design provides support to all contributors in the design and implementation of a clinical trial protocol with patient partners and is organized into four steps (Table 5). Consistent with other how-to guides, the first step of the guide is “Preparation for partnership and patient engagement in clinical development,” followed by “*Building a partnership*,” which includes an end-of-section checklist to aid implementation. Step 3 is “PE in the Clinical Trial Protocol Design,” which has practical recommendations for the collection and analysis of patient insights and, importantly, for providing feedback from patients. It also provides a breakdown of the typical elements of a clinical trial protocol, along with descriptions and examples of relevant questions that can be posed to patient partners and suggests where patients can contribute to protocol design. Step 4 focuses on the feedback and follow-up that should be incorporated alongside the dissemination of results or communication about the project externally. This step aims to support and maintain relationships with partners and contributors after the project formally ends, enhancing future collaborations and increasing the efficiency and impact of PE activities.

**Table 5:** Summary of How-To Guide for PE in clinical trial protocol design

Section	Description and purpose/rationale
STEP 1: Preparation for partnership and patient engagement in clinical development	*Focuses on considerations proposed for sponsors and patient partners before the collaboration starts* Provides practical guidance on how to: ● Establish a collaborative approach by understanding patients’ willingness and abilities to contribute to the clinical trial protocol design and implementation of a clinical trial for the treatment of the disease/condition with which they live ● Assess the level of interest and expectations from patients and sponsors for the collaboration ● Prepare all partners for an impactful collaboration. For example, by providing capacity building activities, such as training and education, on clinical trial protocols and their development; agreeing on common objectives, expectations and time investment required from patients ● Establish the foundation for a long-term, collaborative patient-clinical researcher relationship
STEP 2: Building a partnership	*Focuses on identifying and connecting with potential partners and preparing for the initial meeting to plan the project* Provides practical guidance on ● When to set up a partnership ● Identifying and reaching out to partners ● Planning the first meeting with partners with the PE quality guidance [16]
STEP 3: Patient engagement in clinical trial protocol design	*Focuses on providing guidance and examples of when and how patients can contribute to protocol design* ● Guidance and considerations for collecting patient insights, analysis, and feedback ● Identification of areas for PE in specific sections of typical clinical trial protocol with examples of key questions and aspects that could be considered in each section (e.g., reflecting patient unmet needs and considering patients’ burden around protocol assessments)
STEP 4: Feedback and follow-up	*Focus on maintaining relationships with patient partners and contributors for future collaborations* ● Opportunity to reflect and learn from the project and to assess project impact, including “soft” outcomes ● Guidance on what the ongoing contact could encompass (e.g., a reunion)

## Discussion

The increasing maturity of the PE landscape in medicines development has led to an increasing number of efforts and initiatives to achieve meaningful PE. As a result of this expansion, a need has been identified [16, 43] to draw on existing good practices and documented examples to provide clear guidance on the practice of PE for all stakeholders. There is also a need to provide in-depth and nuanced guidance for specific high-priority PE activities to drive and support practical implementation of PE in medicines development. Crucially, achieving this level of detailed guidance requires input and insights from individual stakeholders with direct experience of PE in these priority activities.

The how-to guides described are novel in several respects. First, they were developed by an international and collaborative set of multi-stakeholder groups. This network included more than 100 individuals from almost 60 organizations, representing a wide range of stakeholder groups across 14 countries. Individuals in the WGs offered diverse and relevant experience and skills required to co-create actionable tools for PE. WG contributors came from different backgrounds, had different levels of experience or maturity in PE (at the individual and/or organizational level), and represented a wide diversity of different perspectives. These characteristics helped to ensure that WG outputs reflect broad perspectives and are relevant across multiple audiences. In addition, the consultation and validation process addressed a mix of audiences and also provided several opportunities to gather wider perspectives along the co-creation journey and not just at one timepoint in the how-to guide co-production. This ensured that each draft how-to guide received the necessary validation to move to the next steps. It also ensured the expansion and diversification of ideas and concepts that could be incorporated into the documents in a coherent and efficient way.

Second, the guides offer a widely applicable framework for engaging patients that is supported by real-life examples of PE in action. They are interconnected and aligned with the phases of the medicines development continuum, providing a seamless set of instructions to involve patients along the entire pathway in the research, development, and delivery of medicines. Thus, the guides are comprehensive, offering information in one set of documents spanning all phases of medicine development. Third, and importantly, each how-to guide is structured in a format that is intuitively organized, consistent, and reproducible; thus, familiarity with one tool is easily translated to another. This feature is expected to facilitate the use of these guides along the medicines development continuum.

Finally, the how-to guides systematically provide an accessible hub of complementary resources that are relevant to the specific activity, such as other guidance, examples of best practice, and key publications. By virtue of these attributes, these guides are expected to advance PE in medicines development by virtue of their content (as well as by facilitating adoption and implementation). This will increase awareness and the education of PE stakeholders.

A public, online, survey-based consultation on each draft how-to guide is underway to gain input from a broader group of stakeholders to capture diverse regional and cultural perspectives and across different levels of PE experience. Respondents are asked general questions about the accessibility and relevance of the how-to guide, in addition to more in-depth questions that focus on content specific to each guide. Public consultation on the guides is being invited across stakeholder groups through PFMD and WG networks and through the PFMD website [44]. There are also accompanying social media, general media, and advertising campaigns to extend the reach and exposure of the consultation. Responses and feedback from the public consultation will be collated, reviewed, and used to refine the how-to guides.

The finalized how-to guides will be made freely available through PFMD’s PEM Suite and shared widely to a large panel of audiences in different settings, ensuring access to diverse patient populations. Deliverables from ongoing projects in other PFMD WGs will also be available in the PEM Suite. These will include resources for PE in regulatory activities, in the post-launch phase, and in the PE training and education repository. There is also a how-to guide about involving patient partners as co-authors in the development and dissemination of PLSs in peer-reviewed publications [45]. The PEM Suite also houses PE Quality Guidance which introduces seven quality criteria to assess PE practices – criteria that are at the core of meaningful PE (https://pemsuite.org/peqg/). The PE Quality Guidance can be used to plan new PE projects or to assess ongoing or completed projects and complements the how-to guides.

In addition to publications and communication in conferences, the intention is to organize virtual educational sessions for organizations and individuals to explore the how-to guides and other WG resources with the contributors who co-created these tools. We encourage those active in PE to pilot the guides and provide feedback that can be used to further refine and improve the guides to deliver meaningful and impactful PE across the medicines continuum. We acknowledge that the guides will not solve all the challenges to implementation of PE but hope that they will help to overcome some key barriers and help to make PE in drug development more consistent.

## Conclusions

We have described the methodology for developing the attributes of a set of how-to guides for incorporating PE into medicines development that can be used by all stakeholders. Co-created guides developed by three PFMD WGs cover PE in early discovery and preclinical phases, COA development, and clinical trial protocol design. The how-to guides form a comprehensive series of actionable and stepwise guides that build from and integrate the PE quality criteria across the medicines continuum. Implementation of these guides should advance the field of PE in bringing new medicines to the market and ultimately benefiting patients.

## Supplementary Information

**Additional file 1: Table S1:** Details of Core Team contributors to Working Group (WG) 1, WG2A and WG2B.

## Data Availability

Not applicable.
