# Supplementary material for: Co-creation of practical “how-to guides” for patient engagement in key phases of medicines development—from theory to implementation
Source: Res Involv Engagem. 2021 Aug 23;7:57. doi: 10.1186/s40900-021-00294-x (PMC8383358; doi:10.1186/s40900-021-00294-x)
Supplement: Supplementary file 1 — Additional file 1: Table S1: Details of Core Team contributors to Working Group (WG) 1, WG2A and WG2B. [file 40900_2021_294_MOESM1_ESM.docx]

**Supplementary Material**

**Table S1:** Details of Core Team contributors to Working Group (WG) 1, WG2A and WG2B

| WG1 (N=13) | WG2A (N=8) | WG2B (N=12) |
| --- | --- | --- |
| Oana Bernard-Poenaru, Servier, France | Ashley Duenas, Evidera, UK | Adit Bassi, GenerationR YPAG, UK |
| Katherine Deane, University of East Anglia, UK | Dagmar Kaschinski, Boehringer Ingelheim, Germany | Angi Gillen, Gilead, US |
| David Feldman, National Kidney Foundation, USA | Dominique Hamerlijnck, EUPATI, Netherlands | Duane Sunwold, National Kidney Foundation, US |
| Grace Fox, The Ottawa Hospital, Canada | Janelle Goins, Gilead , US | Janine Ann Reed, retired nurse, National Kidney Foundation and Alport Syndrome Foundation, US |
| Gorbenko Oleksandr, Ipsen Pharma, UK | Janet Peterson, Takeda, US | Jeanette Ryan, GSK, UK |
| Jim Hartke, Gilead, USA | Jessica Scott, Takeda, US | Jennifer Preston, eYPAGNet, UK |
| Nick Hicks, Commutateur Advocacy Communication, France | Laure Delbecque, Lilly, Belgium | Marta Garcia, Servier, UK |
| Vivian Larsen, Takeda, USA | Paola Kruger, EUPATI, Italy | Olga Zvonareva, Maastricht University, Netherlands |
| Benjamin Missbach,  Ludwig Boltzmann Gesellschaft, Austria |  | Rob Camp, EURORDIS, Spain |
| Claire Nolan, Charities Research Involvement Group, UK |  | Ronella Grootens, Dutch Clinical Research Foundation, Netherlands |
| Natasha Ratcliffe, Parkinson’s UK, UK |  | Severine Wollenschneider, F. Hoffman-LaRoche, Switzerland |
| Carole Scrafton, Fibro Flutters, UK |  | Thierry Escudier, Pierre Fabre, France |
| Merlin Williams, Executive Insights, Switzerland |  |  |

*Core team members of the WG were defined as those who were actively involved in most aspects of each activity from conceptualization and design to co-development and delivery.*
